# Rapid and sensitive detection of waterfowl mycoplasmas using TaqMan assays

**DOI:** 10.1371/journal.pone.0288066

**Published:** 2023-07-14

**Authors:** Edina Nemesházi, Enikő Wehmann, Dénes Grózner, Dorottya Sára Nagy, Áron Botond Kovács, Dorottya Földi, Zsuzsa Kreizinger, Miklós Gyuranecz

**Affiliations:** 1 Veterinary Medical Research Institute, Eötvös Loránd Research Network, Budapest, Hungary; 2 MolliScience Kft., Biatorbágy, Hungary; 3 University of Veterinary Medicine Budapest, Budapest, Hungary; Ain Shams University Faculty of Agriculture, EGYPT

## Abstract

Waterfowl-specific mycoplasmas cause significant economic losses worldwide. However, only limited resources are available for the specific detection of three such bacteria, *Mycoplasma anatis*, *M*. *anseris* and *M*. *cloacale*. We developed species-specific TaqMan assays and tested their reliability across 20 strains of the respective target species as well as 84 non-target avian bacterial strains. Furthermore, we analysed 32 clinical DNA samples and compared the results with those of previously published conventional PCRs. The TaqMan assays showed 100% specificity and very high sensitivity, enabling the detection of target DNA as low as either 10 or 100 copies/μl concentration, depending on the assay. Importantly, we found that while the here developed TaqMan assays are reliable for species-specific detection of *M*. *anatis*, the previously published conventional PCR assay may give false positive results. In conclusion, the new assays are reliable, sensitive and suitable for clinical diagnostics of the target species.

## Introduction

Mycoplasmas are cell wall-less bacteria with a small genome, showing high specificity for their host taxa. The human pathogen mycoplasmas can cause significant challenge for the public health, while the species with veterinary relevance may generate threat for large-scale animal production worldwide. Besides their obvious significance in agriculture, veterinary mycoplasmas may pose ecological importance as well. *Mycoplasma anatis*, *M*. *anseris*, *M*. *cloacale* and *M*. *anserisalpingitidis* are four important waterfowl-specific pathogens. While *M*. *anatis* predominantly infects ducks, the other three species mainly affect geese, and most of these mycoplasma species have also been reported in wild birds [1–5]. These four bacterial species can cause genital and cloacal inflammations, pathological lesions, respiratory and neurological symptoms and reduced egg production as well as increased embryo mortality in waterfowl, resulting in huge economic losses [6, 7]. The waterfowl mycoplasmas can be transmitted both directly and indirectly, horizontally and vertically [4, 8], and some animals can be asymptomatic carriers [9]. Clinical manifestation of mycoplasmosis may be stress related (e.g. inadequate housing conditions or presence of other infectious diseases) and show seasonality (e.g. increase in the egg laying period) [6, 10].

Due to their clinical and economic significance, rapid and accurate diagnosis of mycoplasmal infections is an important challenge. Species-specific identification of the four waterfowl-specific mycoplasmas by conventional PCRs only recently has become available [11]. TaqMan real-time PCR systems are highly specific, and significantly reduce the time required for pathogen identification compared to conventional PCR methods [12]. Taking advantage of these benefits can be useful for clinical diagnostics as well as molecular ecology studies. However, real-time PCR assay has only been established for the identification of *M*. *anserisalpingitidis* [13] and not the other three waterfowl-specific species. Therefore, the aim of the study was to develop species-specific TaqMan assays for *M*. *anseris*, *M*. *anatis* and *M*. *cloacale* to enable reliable and time-efficient detection of their infections.

## Materials and methods

We designed TaqMan primers and probes based on annotated full genomes (NCBI accession numbers: *M*. *anatis*: NZ_CP030141; *M*. *anserisalpingitidis*: NZ_CP041663, NZ_CP041664, NZ_CP042295, NZ_CP083178, NZ_CP082234; *M*. *cloacale*: NZ_CP030103; *M*. *anseris*: NZ_CP030140; note that we follow the conventional nomenclature, see [14]) and on further publicly available whole genome sequences (31 *M*. *anatis* and 110 *M*. *anserisalpingitidis* strains; NCBI Bioprojects: PRJNA856868, PRJNA602215, PRJNA682526, PRJNA856806, PRJNA650261 and PRJNA602206). For this purpose, we analysed the sequences of a total of 28 house-keeping genes that seemed to possess species-specific sequences (S1 Table). The genes were selected either based on the work of Grózner and co-workers [11] or were chosen from the mycoplasmas’ minimal genome set prioritizing the genes with known function and high Simpson’s index of diversity. Based on the close relationship between *M*. *anatis* and *M*. *anserisalpingitidis* genes that were only present in *M*. *anatis* were favoured. For each gene where it was applicable, sequences of *M*. *anatis*, *M*. *anseris*, *M*. *cloacale* and *M*. *anserisalpingitidis* were aligned in Geneoius version 10.2.6 [15]. Primers and probes were designed by the Genescript TaqMan primers and probe design tool (https://www.genscript.com/tools/real-time-pcr-taqman-primer-design-tool) followed by manual optimization. The species-specific primers and probes were selected to be applied under the same temperature profile.

The specificity of the probes and the primer pairs was checked *in silico* using NCBI BLAST NT algorithm (https://blast.ncbi.nlm.nih.gov/Blast.cgi), and the NCBI primer-BLAST tool (NR database, https://www.ncbi.nlm.nih.gov/tools/primer-blast/), respectively. We applied no taxonomic restrictions in the BLAST NT search, and used the primer-BLAST tool (which requires a list of organisms to check) focusing on a broad taxonomic range: mycoplasmas (taxid: 31969), Mycoplasmoidales (taxid: 2790996), Procaryotae (taxid:2157), Aves (taxid: 8782) and, to minimise contamination potentially arising from sample handling, Homo sapiens (taxid: 9606). The primers and probes for the final assays of each target species are shown in Table 1. To test the species specificity of each set of primers and probes, we analysed 20 strains of each of the target species (*M*. *anseris*, *M*. *anatis* or *M*. *cloacale*) (S2 Table), 20 strains of *M*. *anserisalpingitidi*s, as well as 25 additional bacteria occurring in bird hosts: *M*. *columbinasale*, *M*. *columbinum*, *M*. *columborale*, *M*. *gallinaceum*, *M*. *gallinarum*, *M*. *gallisepticum*, *M*. *gallopavonis*, *M*. *imitans*, *M*. *iners*, *M*. *iowae*, *M*. *meleagridis*, *M*. *synoviae*, *Acholeplasma laidlawii*, *Avibacterium paragallinarum*, *Bordetella avium*, *Campylobacter jejuni*, *Clostridium perfringens*, *Erysipelothrix rhusiopathiae*, *Escherichia coli*, *Gallibacterium anatis*, *Pasteurella multocida*, *Riemerella anatipestifer*, *Salmonella sp*., *Staphylococcus aureus* and *Streptococcus gallolyticus*. Therefore, each TaqMan system was tested on 20 target and 85 non-target strains (for example, when *M*. *anseris* was the target, the non-target set of samples consisted of 20 *M*. *anatis*, 20 *M*. *cloacale*, 20 *M*. *anserisalpingitidi*s and 25 other avian bacterium strains). DNA was extracted from each strain using Promega ReliaPrep^TM^ gDNA Tissue Miniprep System (Promega Corp., Madison, WI, USA) following the manufacturers’ protocols. Real-time PCRs were carried out on a Bio-Rad C1000 Touch™ Thermal Cycler, CFX96^TM^ Real-Time System (Bio-Rad Inc., Hercules, CA, USA) with the following settings: initial denaturation at 95°C for 2 minutes followed by 40 cycles of two alternating steps: 5 sec at 95°C and 20 sec at 60°C. Each reaction (except for the *M*. *cloacale*-specific Mclo-*deo*C) took place in a reaction mixture containing 6 μl qPCRBIO Probe Mix No-ROX (2x, PCR Biosystems Inc., Wayne, PA, USA), 0.4 μl forward primer (10 μM), 0.4 μl reverse primer (10 μM), 0.2 μl probe (10 μM) and 2 μl genomic DNA, complemented with nuclease-free water to reach a final volume of 12 μl. PCR mixture for Mclo-*deoC* contained 0.8 μl of the forward primer. Primers and probes are shown in Table 1. Sensitivity of the TaqMan assays were tested by using 2 ul of 10^6^−10^0^ magnitude of template copy number/μl of the type strains of the respective target species (*M*. *anatis* NCTC 10156: NZ_CP030141, *M*. *anseris* ATCC 49234: NZ_CP030140, *M*. *cloacale* NCTC 10199: NZ_CP030103). For this, copy numbers were calculated from DNA concentrations measured by Nanodrop 2000 Spectrophotometer (Thermo Fisher Scientific Inc., Waltham, MA, USA) and the respective genome sizes of the species (*M*. *anatis*: 956 094 bp, *M*. *anseris*: 744 596 bp, *M*. *cloacale*: 661 755 bp), using an online tool (https://cels.uri.edu/gsc/cndna.html), then tenfold dilutions in nuclease-free water were applied. We calculated the limit of detection (LOD), as the lowest copy number of genomic DNA that could be detected in at least 95% of the repeated sensitivity tests, with the threshold being set to 100 RFU. We also calculated the correlation coefficient (R^2^), the slope and the reaction efficiency (E, where 100% means that the amount of target is doubled with each cycle) for each of these tests using the Standard Curve chart in the Bio-Rad CXF Maesto 1.1 software (version 4.1.2433.1219, Bio-Rad Laboratories, Hercules, CA, USA).

**Table 1:** Primers and probes designed for species-specific identification of *M*. *anatis*, *M*. *anseris* and *M*. *cloacale*.

Species	Gene	Name ^1^	Sequence (5’ -> 3’)	Amplicon size
*M*. *anatis*	*Cdd*	Mana-*cdd*-F	CTTCAGGTTTATGTGCT	
		Mana-*cdd*-R	GTATTTCTTTAAAACTTCCAAC	81 bp
		Mana-*cdd*-P	*FAM* - TTATTTGGTTCTGTAGCTCGTGGA –*IBFQ*	
	*ylxR*	Mana-*ylxR*-F	CTATTTGAAAAATGACCCTG	122 bp
		Mana-*ylxR*-R	CCTCCATTAATTCGTTATAAATC	
		Mana-*ylxR*-P	*FAM* - TGCTTTAATTTTAATGAACTGAGACC –*IBFQ*	
*M*. *anseris*	*dnaN*	Mans-*dnaN*-F	GAAGCAGAAGATAATCAAATTAC	205 bp
		Mans-*dnaN*-R	AATCAGTGGCAATTAACATAG	
		Mans-*dnaN*-P	*FAM -* TTATTAAGGTTAGTGCTGATGCTGAA *–IBFQ*	
*M*. *cloacale*	*deoC*	Mclo-*deoC*-F	GATTATGAAAGC**D**AA**Y**GCAA ^2^	103 bp
		Mclo-*deoC*-R	TCTATTAGCACCTAATTCAACC	
		Mclo-*deoC*-P	*FAM -* AATTAAAACCGGAGATGATGCAATT –*IBFQ*	

^1^ The purpose of each oligonucleotide is indicated by the ending of its name: forward primer (F), reverse primer (R) or probe (P).

^2^ Degenerated nucleotide positions are highlighted in bold.

To demonstrate the reliability of the developed TaqMan assays in practice, we validated each assay on a total of 32 clinical samples from six counties of Hungary (Baranya, Szabolcs-Szatmár-Bereg, Csongrád, Hajdú-Bihar, Nógrád, Borsod-Abaúj-Zemplén; Table 2) which were screened for the presence of the target species using the conventional species-specific PCR systems as well [11]. After initial screening with the Mclo-*deoC* system, some clinical samples gave false negative results. Therefore, Sanger sequences from three clinical samples (cl 18, 22, and 25; NCBI accession numbers: OP977968-OP977970) were obtained at Macrogen Europe (Amsterdam, The Netherlands) from the 595-bp long product of the following PCR reaction in 25 μl total volume: 0.25 μl GoTaq (5U/μl; Promega Inc., Madison, WI), 5 μl 5X Green GoTaq Flexi Buffer, 2 μl MgCl_2_ (25 mM; Thermo Fisher Scientific Inc., Waltham, MA), 0.5 μl dNTP (10 mM; Qiagen Inc., Hilden, Germany), 2 μl forward primer (10 μM; 5’ TTATTAAGCCCAGAAGCACT 3’), 2 μl reverse primer (10 μM; 5’ TTACTGATTTCGACATACCT 3’), 11.25 μl nuclease-free water and 2 μl genomic DNA. The temperature profile of this PCR started with an initial denaturation step at 95°C for 5 minutes that was followed by 35 cycles of 30 sec at 95°C, 30 sec at 58°C and 40 sec at 72°C, and ended with a final extension step at 72°C for 5 minutes. Based on the gained sequences, the forward primer was optimised by adding two degenerate nucleotides (see Table 1).

**Table 2:** Detection of *M*. *anatis* (‘Mana’), *M*. *anseris* (‘Mans’) and *M*. *cloacale* (‘Mclo’) in clinical samples with conventional PCR systems [11] and the TaqMan assays.

Sample	Host	Source ^2^	Year	Conventional PCR				TaqMan PCR (Cq values) ^3^			
				Mana-*dna*X	Mans-*pcr*A	Mclo-*dna*X	Mana-*cdd*		Mana-*ylx*R	Mans-*dna*N	Mclo-*deo*C
cl 1	goose	cloaca s.	2015	-	+	+	-		-	32.8	31.8
cl 2	goose	cloaca s.	2016	+	+	+	-		-	33.9	28.9
cl 3	goose	sperm	2016	+	-	+	-		-	-	37.5
cl 4	goose	sperm	2016	+	-	+	-		-	-	33.4
cl 5	goose	phallus l.	2016	-	-	-	-		-	-	-
cl 6	goose	trachea	2016	-	-	-	-		-	-	-
cl 7	duck	cloaca s.	2016	-	-	-	-		-	-	-
cl 8	duck	phallus l.	2016	-	-	-	-		-	-	-
cl 9	goose	cloaca s.	2017	-	+	+	-		-	36.0	27.7
cl 10	goose	cloaca s.	2017	-	-	+	-		-	-	28.1
cl 11	goose	cloaca s.	2017	-	-	+	-		-	-	27.2
cl 12	goose	cloaca s.	2017	-	+	+	-		-	28.9	21.3
cl 13	goose	cloaca s.	2017	-	-	+	-		-	-	21.7
cl 14	goose	cloaca s.	2017	-	+	+	-		-	29.4	28.1
cl 15	goose	cloaca s.	2017	-	+	-	-		-	21.2	-
cl 16	goose	sperm	2017	-	+	+	-		-	20.5	25.1
cl 17	goose	cloaca s.	2017	-	+	+	-		-	36.8	28.6
cl 18 ^1^	goose	cloaca s.	2017	-	-	+	-		-	-	30.6
cl 19	goose	cloaca s.	2022	-	-	-	-		-	-	-
cl 20	goose	cloaca s.	2022	-	-	-	-		-	-	-
cl 21	goose	cloaca s.	2022	-	-	-	-		-	-	-
cl 22 ^1^	goose	cloaca s.	2022	-	+	+	-		-	31.8	26.1
cl 23	goose	cloaca s.	2022	-	+	+	-		-	29.0	25.0
cl 24	goose	cloaca s.	2022	-	+	+	-		-	32.3	26.4
cl 25 ^1^	goose	cloaca s.	2022	-	+	+	-		-	34.8	26.01
cl 26	duck	cloaca s.	2019	+	-	-	35.4		34.7	-	-
cl 27	duck	cloaca s.	2019	+	-	+	23.5		22.8	-	27.8
cl 28	duck	cloaca s.	2019	+	-	+	24.6		24.5	-	28.6
cl 29	duck	cloaca s.	2019	+	-	+	23.6		23.5	-	24.3
cl 30	duck	cloaca s.	2019	+	-	+	25.5		24.9	-	29.7
cl 31	NA	cloaca s.	2022	+	-	-	21.7		22.1	-	-
cl 32	NA	cloaca s.	2022	+	-	-	23.0		22.7	-	-

^1^ NCBI accession numbers: OP977968-OP977970.

^2^ Abbreviations: cloaca swab (cloaca s.), phallus lymph (phallus l.)

^3^ The abbreviation Cq stands for quantification cycle.

## Results

**Fig 1:**
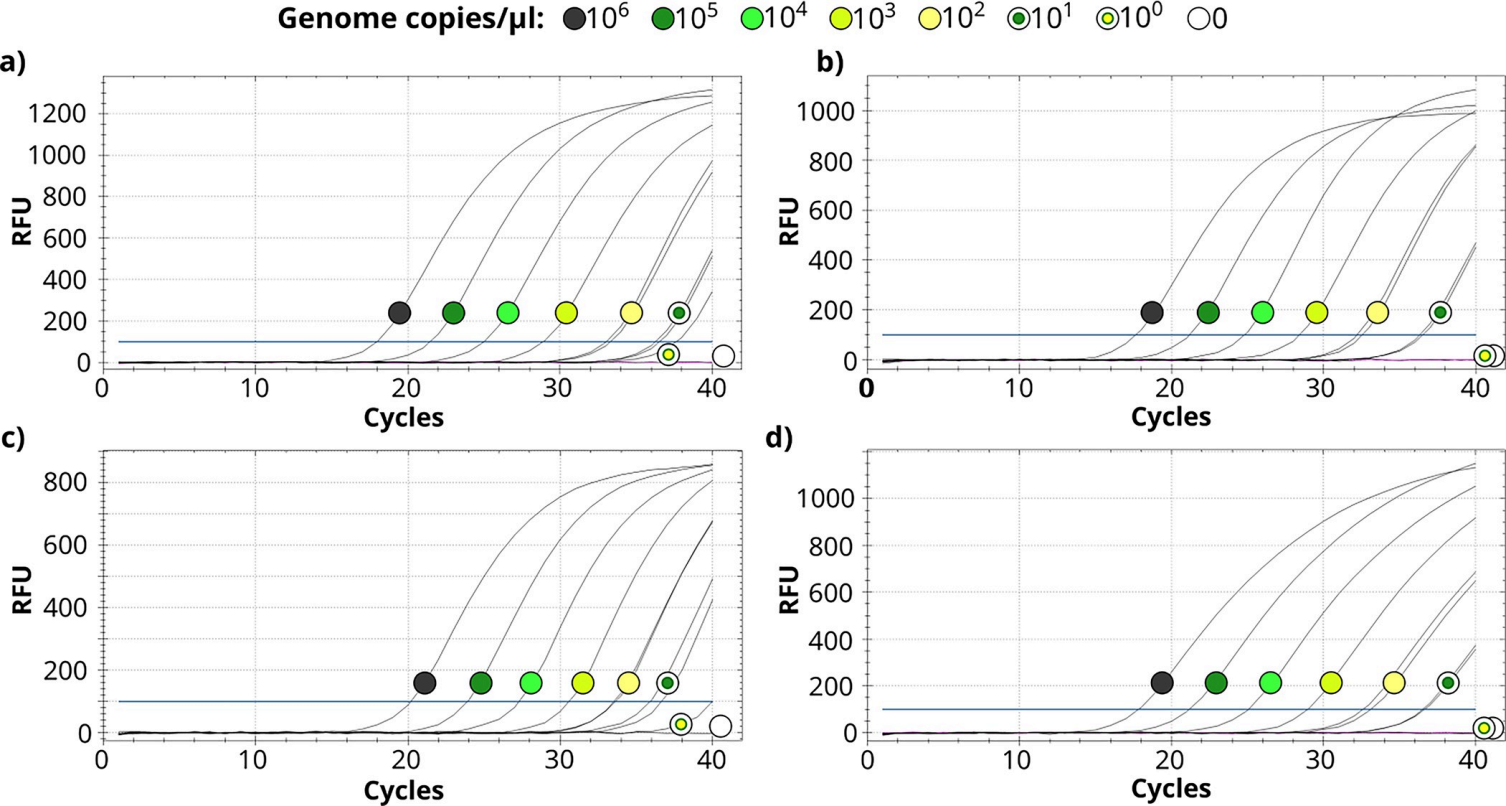
Sensitivity of each species-specific TaqMan system: Mana-*cdd* (a) and Mana-*ylxR* (b) for *M*. *anatis*, Mans-*dnaN* for *M*. *anseris* (c) and Mclo-*deoC* for *M*. *cloacale* (d). Duplicate reactions were performed between 10^0^ and 10^3^ copy numbers. RFU: relative fluorescence units. The horizontal line at 100 RFU indicates the threshold for positivity. For results of further sensitivity tests repeated on different days see S5 Table.

We developed and tested four TaqMan systems (Table 1): one for *M*. *anseris* (Mans-*dnaN*; DNA polymerase III subunit beta gene), one for *M*. *cloacale* (Mclo-*deoC*; deoxyribose-phosphate aldolase gene) and two for *M*. *anatis* (Mana-*cdd* and Mana-*ylx*R; cytidine deaminase and YlxR family protein genes, respectively). The *in silico* tests of probes and primer pairs indicated overall high species specificity, as the species identity of potential false matches (if any) always differed between the primer pair and the probe of each TaqMan assay. See the first six results (as sorted by E-value) of each NCBI BLAST NT search in S3 Table, and all results identified by NCBI primer-BLAST in S4 Table. According to the calculated LOD values of the repeated sensitivity tests (Fig 1, S5 Table), the Mana-*ylx*R and Mclo-*deo*C TaqMan assays detected as low as 10 target copies per μl, while the Mana-*cdd* and Mans-*dna*N assays detected the magnitude of 10^2^ target copies per μl confidently. The values of R^2^ ranged between 0.977 and 0.999, the slopes ranged between -3. 189 and -4.042, while the E values ranged between 76.8% and 105.8% (S5 Table). In the specificity tests with each of the four TaqMan systems, all 20 target strains were found positive, while no false positives were detected among the 85 non-target strains (S2 Table).

Results of the TaqMan assays corresponded with those of the conventional species-specific PCRs across all 32 clinical samples for *M*. *cloacale* and *M*. *anseris* (Table 2). By contrast, three out of 10 samples that were positive for *M*. *anatis* based on the conventional PCR (targeting the *dna*X gene; annotated as DNA polymerase III subunit gamma/tau in *M*. *anatis* genomes) were negative with both Mana-*cdd* and Mana-*ylxR*. Certain *M*. *anserisalpingitidis* strains examined in the present study possessed *M*. *anatis*-like sequence on the *dnaX* gene (MYCAV 205 and MYCAV 264, genomes published as part of NCBI BioProject PRJNA602215, the second being isolated from clinical sample cl 3), and the *dnaX*-based *M*. *anatis*-specific PCR turned out to be positive for these species, while no basis for cross-amplification was detected for the *cdd* and *ylx*R markers. Similarly, clinical samples cl 2—cl 4 in Table 2 showed incongruent results with the conventional and real-time PCR assays.

## Discussion

Stress can facilitate the clinical manifestation of mycoplasmosis [8]. In captivity, for example, inadequate housing such as high bird density, limited space, limited water supply and the presence of mixed-aged fowls increase anxiety in waterfowl. Moreover, sexual activity, extensive egg production and animal transportation are stress factors as well. It has been hypothesised that as the frequency of hot days and nights will increase with climate change, disease outbreaks may also become more frequent in some waterfowl populations [16]. Waterfowl stocks can carry *M*. *anatis*, *M*. *anseris* and *M*. *cloacale* infections unnoticed, hence the monitoring of these pathogens is neglected. However, because of the above-mentioned processes, these pathogens may have a more significant negative impact on economics in the future [6], and may amplify the severity of co-infections with further pathogens.

In the present study, the results suggested 100% species specificity of all four TaqMan assays developed here for three waterfowl pathogens. Furthermore, sensitivity of these tests was high, with a magnitude of 10^2^ genome copies per microliter being detectable with each system, and as low as 10^1^ genome copies being detectable with the Mana-*ylx*R and Mclo-*deo*C. The assays were also suitable for detecting the presence of *M*. *anatis*, *M*. *anseris* and *M*. *cloacale* in clinical samples from various sources, including cloaca swabs, sperm and phallus lymph samples. Results of the TaqMan assays concurred with those of the previously published PCR markers when the presence of *M*. *cloacale* and *M*. *anseris* was assessed in clinical samples [11]. Because the TaqMan assays are more sensitive compared to the previously published PCR assays (10^1^ or 10^2^ *versus* 10^3^), the new tests are more suitable for examining clinical samples. Furthermore, as the supposedly *M*. *anatis*-specific *dna*X PCR target [11] gave false positive results in some *M*. *anserisalpingitidis* strains, only the TaqMan assays are reliable for the detection of *M*. *anatis*. Similarities between the genomes of *M*. *anatis* and *M*. *anserisalpingitidis* likely stem from their close evolutionary relationships, because these species seem to be recent descendants of a common ancestor [17, 18]. Therefore, the usage of multiple assays is advisable for differentiation between these two species. According to the presented results, the two new *M*. *anatis*-specific TaqMan systems developed here may fulfil this purpose.

The new diagnostic TaqMan assays enable species identification with high sensitivity and specificity while notably reducing the time required for diagnosis compared to the methods that have been available so far for *M*. *anatis*, *M*. *anseris* and *M*. *cloacale* [1, 5, 8, 11]. These assays are expected to be suitable for infection diagnostics and monitoring in both farm animals and wild populations of waterfowl [1–5]. With these sensitive assays, even symptomless carriage of pathogens may be identified, enabling future studies to reveal the prevalence of these bacteria which could contribute to our understanding on their global epidemiology. Because all four TaqMan systems run under the same thermal cycling profile, which takes less than one hour, they provide a very convenient, time-efficient, highly sensitive and reliable alternative to the conventional, often time-consuming and sometimes less reliable diagnostic methods.

## Supporting information

S1 Table Genes considered for TaqMan-primer development in each species. (XLSX)

S2 Table Avian bacterium strains used for testing species-specificity of TaqMan assays. (XLSX)

S3 Table The first six results of NCBI BLAST NT of each TaqMan probe, as sorted by E-value. (XLSX)

S4 Table All results of NCBI primer-BLAST of each TaqMan primer pair. (XLSX)

S5 Table Statistics calculated for repeated sensitivity tests of each TaqMan assay. Note that the numbers of replicates differed between tests. Overall LOD indicates the lowest magnitude of target DNA copies that was successfully amplified by all sensitivity tests for each assay.
(XLSX)
